# Health-related heterogeneity in brain aging and associations with longitudinal change in cognitive function

**DOI:** 10.3389/fnagi.2022.1063721

**Published:** 2023-01-04

**Authors:** Jo Wrigglesworth, Joanne Ryan, Phillip G. D. Ward, Robyn L. Woods, Elsdon Storey, Gary F. Egan, Anne Murray, Sara E. Espinoza, Raj C. Shah, Ruth E. Trevaks, Stephanie A. Ward, Ian H. Harding

**Affiliations:** ^1^School of Public Health and Preventive Medicine, Monash University, Melbourne, Vic, Australia; ^2^Monash Biomedical Imaging, Monash University, Clayton, Vic, Australia; ^3^Australian Research Council Centre of Excellence for Integrative Brain Function, Clayton, Vic, Australia; ^4^Hennepin Healthcare and Berman Center for Outcomes & Clinical Research, Hennepin Healthcare Research Institute, Minneapolis, MN, United States; ^5^Department of Medicine, Division of Geriatrics, Hennepin Healthcare, University of Minnesota, Minneapolis, MN, United States; ^6^Division of Geriatrics, Gerontology & Palliative Medicine, Barshop Institute for Longevity and Aging Studies, University of Texas Health Science Center, Houston, TX, United States; ^7^Geriatric Research, Education & Clinical Center, South Texas Veterans Health Care System, San Antonio, TX, United States; ^8^Department of Family & Preventive Medicine and the Rush Alzheimer’s Disease Center, Rush University Medical Center, Chicago, IL, United States; ^9^Centre for Healthy Brain Ageing (CHeBA), University of New South Wales, Sydney, NSW, Australia; ^10^Department of Geriatric Medicine, Prince of Wales Hospital, Randwick, NSW, Australia; ^11^Department of Neuroscience, Central Clinical School, Monash University, Melbourne, VIC, Australia

**Keywords:** brain aging, cognitive, brain-predicted age, physical health outcomes, neuroimaging

## Abstract

**Introduction:**

Neuroimaging-based ‘brain age’ can identify individuals with ‘advanced’ or ‘resilient’ brain aging. Brain-predicted age difference (brain-PAD) is predictive of cognitive and physical health outcomes. However, it is unknown how individual health and lifestyle factors may modify the relationship between brain-PAD and future cognitive or functional performance. We aimed to identify health-related subgroups of older individuals with resilient or advanced brain-PAD, and determine if membership in these subgroups is differentially associated with changes in cognition and frailty over three to five years.

**Methods:**

Brain-PAD was predicted from T1-weighted images acquired from 326 community-dwelling older adults (73.8 ± 3.6 years, 42.3% female), recruited from the larger ASPREE (ASPirin in Reducing Events in the Elderly) trial. Participants were grouped as having resilient (n=159) or advanced (n=167) brain-PAD, and latent class analysis (LCA) was performed using a set of cognitive, lifestyle, and health measures. We examined associations of class membership with longitudinal change in cognitive function and frailty deficit accumulation index (FI) using linear mixed models adjusted for age, sex and education.

**Results:**

Subgroups of resilient and advanced brain aging were comparable in all characteristics before LCA. Two typically similar latent classes were identified for both subgroups of brain agers: class 1 were characterized by low prevalence of obesity and better physical health and class 2 by poor cardiometabolic, physical and cognitive health. Among resilient brain agers, class 1 was associated with a decrease in cognition, and class 2 with an increase over 5 years, though was a small effect that was equivalent to a 0.04 standard deviation difference per year. No significant class distinctions were evident with FI. For advanced brain agers, there was no evidence of an association between class membership and changes in cognition or FI.

**Conclusion:**

These results demonstrate that the relationship between brain age and cognitive trajectories may be influenced by other health-related factors. In particular, people with age-resilient brains had different trajectories of cognitive change depending on their cognitive and physical health status at baseline. Future predictive models of aging outcomes will likely be aided by considering the mediating or synergistic influence of multiple lifestyle and health indices alongside brain age.

## Introduction

Aging is a complex biological phenomenon, characterized by the gradual accumulation of molecular, cellular and tissue damage (Lopez-Otin et al., 2013). The body’s inability to repair itself precedes a decline in physiological functions, including sensory, motor, and cognitive functions that are important for maintaining independence and quality of life (Lopez-Otin et al., 2013; World Health Organization, 2015). The brain is susceptible to the effects of aging, undergoing many structural and functional changes over the lifespan. Most widely recognized is brain atrophy (the loss of tissue volume), which has been associated with a decline in cognitive function (Cabeza et al., 2018; Grajauskas et al., 2019). However, there is considerable diversity in the rate of aging, which is influenced by genetic and environmental factors.

Modelling ‘brain age’ has deepened our understanding of the interindividual differences in brain aging. This includes ‘brain age’, a measure of biological age that is predicted from neuroimaging at the individual level relative to a normative model. A person’s brain-predicted age difference (brain-PAD) represents the deviation of brain age from the normal aging trajectory. An older brain age relative to chronological age is considered a sign of ‘*advanced*’ brain aging (i.e., greater age-related brain atrophy), and has been linked with a lower cognition and a greater risk of dementia (Franke et al., 2010; Elliott et al., 2021). Conversely, a brain appearing younger than expected relative to one’s chronological age reflects ‘*resilient*’ brain aging, and has been predictive of better physical fitness and cognitive function (Kolbeinsson et al., 2020; Sanders et al., 2021). A number of brain age algorithms have been developed, including a model by Cole et al. (2018). This uses voxel-level measures of grey matter (GM), white matter (WM) and cerebrospinal fluid (CSF) volume to provide a single estimate of brain age measured across the whole brain. Using this model, we have found an association between advanced brain aging and poor cognitive processing (Wrigglesworth et al., 2022a), and identified sex differences in the change in brain aging over time (Wrigglesworth et al., 2022b).

As a surrogate measure of brain health, brain-PAD has provided useful insights into individual differences in biological aging trajectories, and their direct relationship with cognition, brain diseases, and other health outcomes (Lowe et al., 2016; Cole et al., 2020; Vidal-Pineiro et al., 2021). However, evidence suggests there may be additional heterogeneity hidden within populations of resilient or advanced brain agers (Eavani et al., 2018). For instance, Eavani et al. (2018) identified five different brain imaging phenotypes in cognitively unimpaired older adults with advanced brain aging, demonstrating differential neuroanatomical substrates, and thus potentially unique pathological pathways, underlying trajectories of unhealthy brain aging. Sub-categorizing people with a resilient or advanced brain-PAD may therefore be an important avenue of inquiry that could provide greater individual specificity in predicting distinct profiles of current and future health status. To address this hypothesis, this study aimed to examine heterogeneity within each category of brain-PAD relative to other health, behavioral or cognitive measures. We examined this question independently for each brain age group (advanced and resilient) to avoid assumptions regarding unique or overlapping outcomes within these qualitatively distinct cohorts. Through the use of data-driven approaches, we first identify healthy elderly people with advanced and resilient brain age from a large community-based cohort, then we identify unique clusters within these advanced and resilient brain age groups based on other health factors, and finally we determine whether these subprofiles are differentially associated with longitudinal change in cognitive function and frailty.

## Materials and methods

### Study participants

This study used neuroimaging data from ASPREE-NEURO (NEURO; Ward et al., 2017), a substudy of the ASPirin in Reducing Events in the Elderly (ASPREE) clinical trial (ASPREE Investigator Group, 2013). Eligible criteria for ASPREE have been published elsewhere (ASPREE Investigator Group, 2013). NEURO recruited 572 ASPREE participants residing in Melbourne and nearby regional Victoria, Australia. Of these participants, 557 healthy volunteers completed a magnetic resonance imaging (MRI) scan at baseline from which brain-PAD could be determined (a median duration of 14 days after randomization into ASPREE), with subsequent scans performed one (*n* = 516) and 3 years later (*n* = 472). A total of 326 participants were eligible for this study as measures of brain aging had met the inclusion criteria (*refer to section ‘Latent Class Analysis for data clustering’*). Participants provided written informed consent to both ASPREE and NEURO, and study procedures were conducted in accordance with institutional guidelines. ASPREE is registered with the International Standard Randomized Controlled Trial Number Register (ISRCTN83772183) and Clinicaltrials.gov (NCT01038583). NEURO is registered with the Australian and New Zealand Clinical Trial Registry (ACTRN12613001313729). The current study was approved by the Monash University Human Research and Ethics Committee (Project ID: 29311).

### Neuroimaging data collection and quality control

Three-dimensional T1-weighted magnetization-prepared rapid gradient echo (MPRAGE) images were acquired using a 3 Tesla Siemens Skyra MR scanner (Siemens Erlangen, German) with a 32-channel head and neck coil, located at Monash Biomedical Imaging in Melbourne, Australia (192 sagittal slices; 1 mm isotropic voxels; FOV = 256 × 240 mm^2^; TR = 2,300 ms; TE = 2.07 ms; TI = 900; flip angle = 9°). Image quality was quantitatively assessed using the MRI Quality Control tool (MRIQC; Esteban et al., 2017), with outliers qualitatively inspected by three study investigators (JW, PW, IHH), as previously described (Wrigglesworth et al., 2022a).

### Brain age estimation

Brain age was estimated using the trained model developed by Cole and colleagues (Cole et al., 2018).^1^ Images were pre-processed using the Statistical Parametric Mapping (SPM12) toolbox (University College London, London, United Kingdom), including segmentation into GM, WM, and CSF, and normalization to the Montreal Neurological Institute (MNI) space using a non-linear registration algorithm (DARTEL; Cole et al., 2015). For each individual, the tissue volume at each voxel was encoded by modulating the spatially normalized GM and WM partial-volume images by the Jacobian determinant of the deformation from subject space to MNI space (Ashburner, 2009). Images were resampled to a voxel size of 1.5 mm, and smoothed using a Gaussian spatial smoothing kernel of 4 mm at full-width-half maximum (Cole et al., 2015).

The normalized images were combined and reduced to 435 principal components previously identified for a training cohort of 3,377 healthy adults (aged 18 to 92 years), sourced from seven publicly available datasets, which cover a range of geographical locations (including Australia, the United States, and United Kingdom), scanner strengths and data acquisitions (refer to for further details; see Footnote 1). Components accounting for 80% of the total variance of chronological age were included as input into a Gaussian process algorithm, and the resulting rotation matrix was used to predict brain age for the NEURO participants.

Brain-PAD represents the deviation of brain age from chronological age. A positive value (i.e., older looking brain relative to one’s chronological age) is considered a sign of advanced brain aging; while negative values (i.e., younger brain age relative to chronological age) indicate resilient brain aging. While our prior findings show no statistically significant correlation between brain-PAD and chronological age (*ρ* = −0.01, *p* = 0.83), and thus no age bias (i.e., models underestimate brain age for older individuals and overestimate brain age for younger people; Wrigglesworth et al., 2022a), we included chronological age as a covariate in models examining the change in cognition and frailty (*refer to ‘Longitudinal prediction of change in cognitive function and frailty’*).

### Lifestyle, cognitive function, and health measures

Included in the latent class analysis (LCA) were measures of cardiometabolic health, lifestyle, cognitive function, and well-being, collected at baseline in the ASPREE clinical trial (McNeil et al., 2017). Binary variables relevant to this study included: (i) ever smoked; (ii) obesity (body mass index >30 kg/m^2^); and (iii) hypertension (systolic and/or diastolic blood pressure (BP) > 140/90, or on treatment for high BP). Additional summary measures of self-reported (iv) physical quality of life (QoL) and (v) mental QoL were assessed using the Short-Form-12 (SF-12; Ware et al., 1996), as previously described (Phyo et al., 2021b), and divided into tertiles; higher scores reflect better QoL. General cognitive status was assessed using the (vi) Modified Mini-Mental State Examination (3MS; Teng and Chui, 1987), and divided into tertiles; higher scores reflect better cognitive function.

### Latent class analysis for data clustering

**Figure 1:**
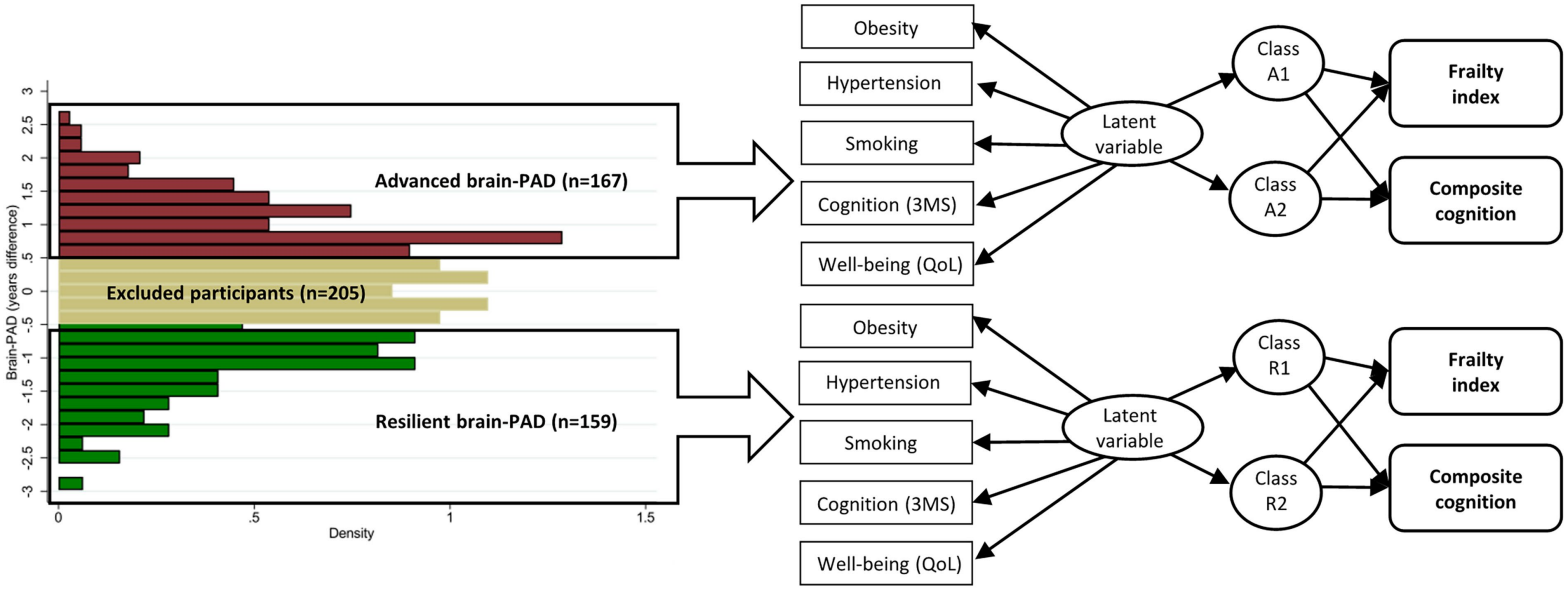
Overview of study methods, including identification of resilient and advanced brain agers, latent class analyses and the association between classes and the change in health outcomes over time. Abbreviations: 3MS = global cognitive function measured using Modified-Mini-Mental State (3MS) examination score (Teng and Chui, 1987); brain-PAD = brain-predicted age difference; QoL = quality of life.

To initially identify unique health clusters of advanced and resilient brain agers the cohort was first divided into ‘advanced’ and ‘resilient’ brain aging groups; defined by a brain-PAD greater or lesser than 0.5 standard deviations from the mean (i.e., those within 0.5 SD of the mean were discarded). Here was an approach to identify participants at the tail ends of the distribution to avoid overlapping and bias attributed to the brain age model or noise inducing image artefacts. Data-driven LCA were then performed to identify subclasses within each of these groups based on the functional, cognitive, and health measures described above (Figure 1).

LCAs were performed using a conventional stepwise approach to identify the optimal number of classes in the data. This involved first fitting a single-class model, then a two-class model, etc., until the next model in the sequence failed to converge (Masyn, 2013; Sinha et al., 2021). Optimal convergence was evaluated using multiple unconditional LCAs, with 100 random starting values (set seed of 15). Several fit indices were used to determine the optimal number of classes, such as the smallest Akaike’s information criterion (AIC) and Bayesian information criterion (BIC; Masyn, 2013). The extent to which the observed variables predicted class membership was evaluated using the average class probability (AvePP; Masyn, 2013). Class assignment was considered adequate when AvePP values were greater than 0.70 (Nagin, 2005). Once the models had been fitted, we assigned participants to their most likely class using their highest-class posterior probability. The assumption of local independence was examined by the strength of the association between two categorical variables, conditional to the latent class, using the Cramer’s V. Associations greater than 0.5 were further evaluated for their impact on the LCA parameters (Sinha et al., 2021).

Classes were described according to how well each measure epitomized each class (homogeneity) and distinguished one class from the other (separation). A class-specific item probability (i.e., the probability that a person in that assigned class will endorse that response; Nylund et al., 2007) less than 0.30, or greater than 0.70, defined a characteristic with a high class homogeneity (Masyn, 2013). Separation was indicated by a statistically significant difference in the relative frequency between classes, using the chi-square test.

To examine whether classes are dependent on brain health, an additional LCA was performed on the total cohort of resilient and advanced brain agers (*n* = 326) using the methods described above. Analyses were performed using Stata software, version 17.0 (StataCorp).

### Longitudinal prediction of change in cognitive function and frailty

To determine whether health clusters of brain aging relate to other measures of aging, we investigated whether class membership was differentially associated with changes in cognition and frailty. Primary endpoints of ASPREE (ASPREE Investigator Group, 2013), including mortality, cardiovascular disease and persistent disability, could not be used due to the small number of events.

Longitudinal change in cognition was quantified using a composite cognitive function score. The composite score was derived using a summed z-score from four individual tests assessing verbal fluency [Controlled Oral Word Association Test (Ross, 2003)], episodic memory [the Hopkins Verbal Learning Test – Revised Delayed Recall task (Benedict et al., 1998)], psychomotor speed [Symbol Digit Modalities Test (Smith, 1982)], and general cognitive status (3MS). The composite approach was adopted to reduce noise and floor/ceiling effects (Jutten et al., 2019; Ryan et al., 2020). A higher score indicates a better global cognitive performance. Cognition was assessed at baseline, year 1, year 3 and year 5.

The frailty index (FI) is derived from 67 health deficit measures, reflecting a range of health conditions, disease indicators, physical disabilities, mental and psychosocial deficits, cognitive function and physical performance (Ryan et al., 2022). Scores are calculated as the sum of all deficits, divided by the number of items available; calculations were performed for participants with data for at least 50 items to avoid bias or a reliance on imputation (Ryan et al., 2022). Total scores range from 0 to 1, with a higher score indicating a greater number of deficits. The FI was measured at baseline, and at annual visits over 3 years. Data from later assessments (i.e., 4-, 5-, 6- and 7-year visits) were not available at the time of calculating FI, therefore could not be included in this study.

Linear mixed models were used to investigate the association between the latent subclasses and the longitudinal rate of change in FI and composite cognitive function. Models were fitted independently for the resilient and advanced brain aging groups, and subsequently the total cohort for our additional analysis. These include the fixed effects of time (i.e., annual visits with a value of 0 [baseline], 1, 3, and 5 years) and exposure (i.e., latent class membership, and a binary variable for defining resilient or advanced brain aging in the additional analysis), along with the interaction between time and exposure to examine the rate of change in study outcomes, and a random intercept and slope. Models were adjusted for chronological age, sex and education.

## Results

### Participant characteristics

**Figure 2:**
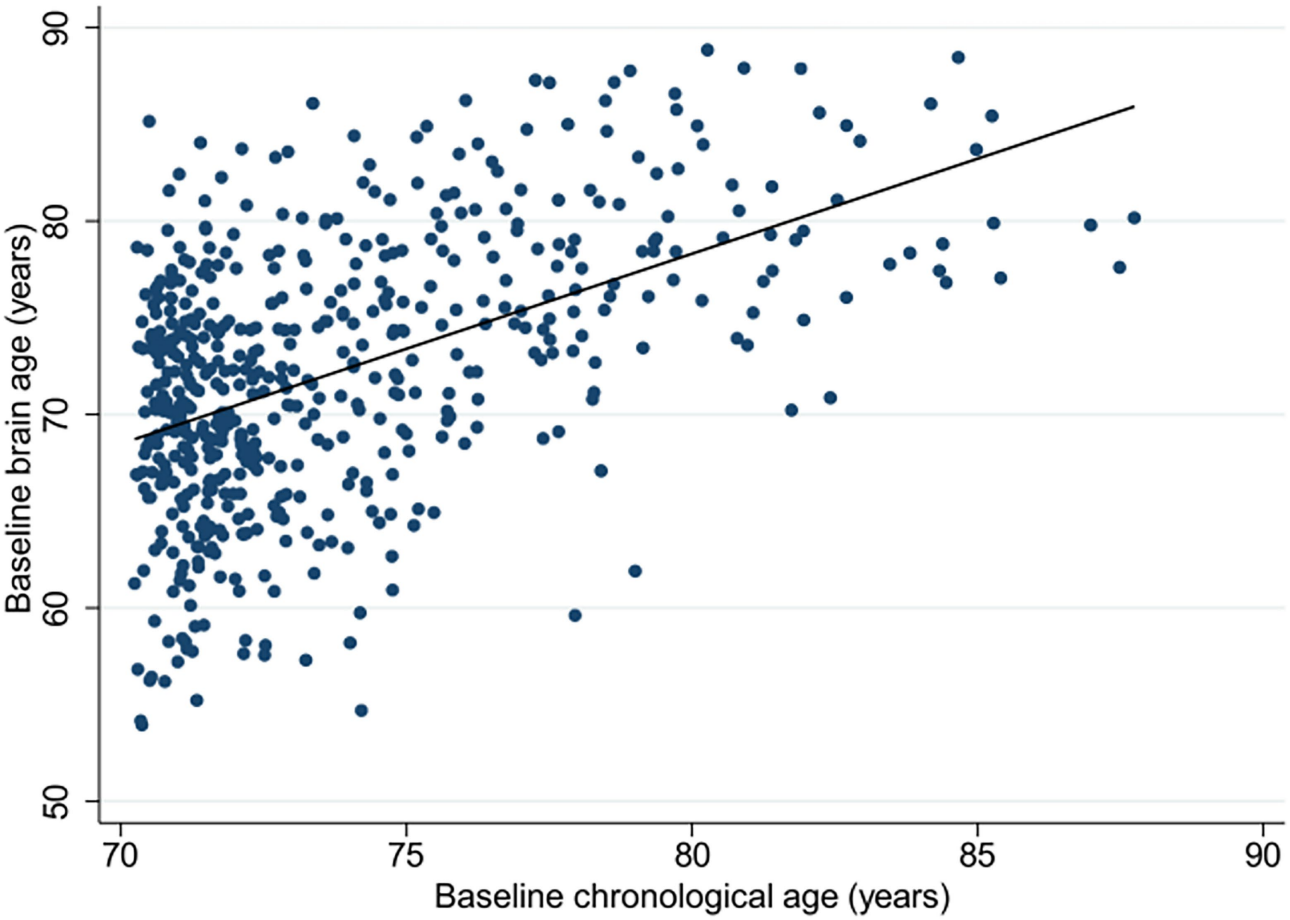
Scatterplot presenting the correlation between the estimated brain age (y-axis) and chronological age (x-axis), measured at baseline for the total NEURO cohort (*n* = 531).

Brain age was estimated for 531 NEURO participants (mean age of 73.8 ± 3.5 years; 48.4% were female), following the exclusion of 26 participants based on inadequate image quality. Here, the estimated brain age had a significant positive correlation with chronological age (*ρ* = 0.46, *p* < 0.0001; Figure 2), and a mean absolute error (MAE) of 4.96. For this study, 159 participants met our criteria for resilient brain aging (mean brain-PAD of -8.7 ± 3.3 years), and 167 for advanced brain aging (mean brain-PAD of 5.15 ± 2.7 years). These subgroups were comparable in all baseline participant characteristics (Supplementary Table 1).

Two hundred and five people within the ‘normal’ range of brain age relative to their chronological age (i.e., brain-PAD was within 0.5 standard deviation from the mean) were excluded. These participants were comparable to our study sample, except there was a higher proportion of women, and higher mean 3MS score (Supplementary Table 1).

### Latent class analysis

**Figure 3:**
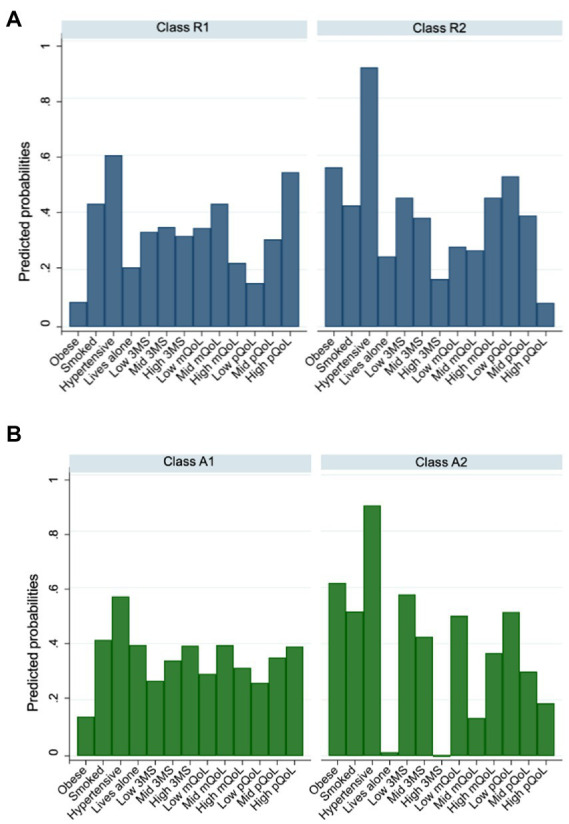
Column graph summarizing item-class probabilities from unconditional latent class models, performed separately for **(A)** resilient **(B)** and advanced brain age groups. Abbreviations: 3MS = global cognitive function measured using Modified-Mini-Mental State (3MS) examination score (Teng and Chui, 1987); mQoL = mental quality of life; pQoL = physical quality of life.

From the latent class analysis, the best solution was two classes for both the resilient and advanced brain age groups (Figure 3; class-specific item probabilities are reported in Supplementary Table 2). For resilient brain aging, selection was based on the model reaching maximal convergence before identification was insufficient. For the advanced brain age group, a maximum identified was three classes, but a two class solution was chosen for its parsimony and preventing model overfitting (Coelho et al., 2019). The precision of class assignment was evaluated, with an AvePP greater than 0.80 and 0.70 in the resilient and advanced brain age groups, respectively, suggesting a low chance of misclassification. Identification of the global maximum was confirmed, and both models showed an adequate absolute fit (resilient: *G^2^* = 294.0, *p* = 1.00; advanced: *G^2^* = 311.1, *p* = 1.00). We identified no strong relationship (as indicated by a Cramer’s V greater than 0.5) between variables within each class, and thus the assumption of local independence was not violated (Supplementary Tables 3, 4; Sinha et al., 2021).

Classes of resilient and advanced brain aging are characterized in Table 1. For the resilient brain age group, a relatively equal proportion were assigned to class R1 (54%) and R2 (47%). Class R1 was characterized by a low prevalence of obesity, smaller likelihood of high (tertile 3) mental QoL, and smaller likelihood of low (tertile 1) physical QoL. Class R2 was characterized by a higher prevalence of hypertension, lower probability of high (tertile 3) general cognitive status and physical QoL, and low to moderate scores (tertile 2) in mental QoL.

**Table 1:** Demographic and lifestyle characteristics of the latent classes, separately for the resilient and advanced brain aging.

	Resilient (*n*=159)			Advanced (*n*=167)		
Characteristics	Class R1 (53.5%)	Class R2 (46.5%)	*p*	Class A1 (67.7%)	Class A2 (32.3%)	*p*
Age group			0.75			0.25
<75	65 (76.5)	55 (74.3)		83 (73.5)	35 (64.8)	
75+	20 (23.5)	19 (25.7)		30 (26.6)	19 (35.2)	
Female gender, n (%)	33 (38.8)	35 (47.3)	0.28	52 (46.0)	18 (33.3)	0.12
<12 years education, n (%)	30 (35.3)	26 (35.1)	0.98	76 (67.3)	30 (55.6)	0.14
Obese, n (%)	**5 (5.9)**	45 (60.8)	<0.0001	**12 (10.7)**	35 (64.7)	<0.0001
Ever smoked, n (%)	37 (43.5)	31 (41.9)	0.84	47 (41.6)	27 (50.0)	0.31
Hypertensive, n (%)	49 (57.7)	**70 (94.6)**	<0.0001	62 (54.9)	**49 (90.7)**	<0.0001
Lives alone, n (%)	**16 (18.8)**	**20 (27.0)**	0.22	47 (41.6)	**0**	<0.0001
3MS overall score, n (%)			0.01			<0.0001
Tertile 1 (78-93)	27 (31.8)	35 (47.3)		**29 (25.7)**	31 (57.4)	
Tertile 2, (94-96)	29 (34.1)	29 (39.2)		38 (33.6)	23 (42.6)	
Tertile 3, (97-100)	29 (34.1)	**10 (13.5)**		46 (40.7)	**0**	
Mental QoL, n (%)			<0.0001			<0.0001
Tertile 1 (28.5-54.7)	31 (36.5)	**19 (25.7)**		**31 (27.4)**	28 (51.9)	
Tertile 2 (54.7-59.1)	38 (44.7)	**18 (24.3)**		48 (42.5)	**5 (9.3)**	
Tertile 3 (59.1-78.8)	**16 (18.8)**	37 (50.0)		34 (30.1)	21 (38.9)	
Physical QoL, n (%)			<0.0001			<0.0001
Tertile 1 (16.2-46.6)	**9 (10.6)**	44 (59.5)		**27 (23.9)**	29 (53.7)	
Tertile 2 (46.8-54.0)	28 (32.9)	27 (36.5)		39 (34.5)	17 (31.5)	
Tertile 3 (54.0-63.7)	48 (56.5)	**3 (4.1)**		47 (41.6)	**8 (14.8)**	

Bold = high class homogeneity, defined by a class-item probability ≤0.30 or ≥0.70 (Masyn, 2013). Hypertensive individuals reporting anti-hypertensive treatment (Class R1: *n* = 26[32.5%]; Class R2: *n* = 49[67.1%]; Class A1: *n* = 38[36.9%]; Class A2: n = 39[72.2%]). Missing data for obesity (advanced: *n* = 1) and anti-hypertensive medication data (resilient: *n* = 6; advanced: *n* = 10) at baseline. Abbreviations: 3MS = global cognitive function measured using Modified-Mini-Mental State (3MS) examination score (Teng and Chui, 1987); QoL = quality of life.

For the advanced brain age group, 68% were assigned to class A1, while the remaining 32% were defined by class A2 (Table 1). The characteristics that defined these two classes were very similar to those for resilient brain agers, with the exception of there being a lower prevalence of people who lived alone in class A2. Classes R1 and R2, and A1 and A2 were comparable in chronological age, sex and education (Table 1).

To examine a dependency of health-related heterogeneity on brain health, we performed an additional LCA on the total cohort (*n* = 326). While a maximum of three classes was observed, the two class model was considered optimal, for reasons previously described for advanced brain agers (i.e., parsimony and overfitting). Results are reported in Supplementary Tables 5, 6. Characteristically, these subclasses largely replicate clusters generated separately for the advanced and resilient subgroups.

### Class association with longitudinal change in cognitive function and frailty

**Figure 4:**
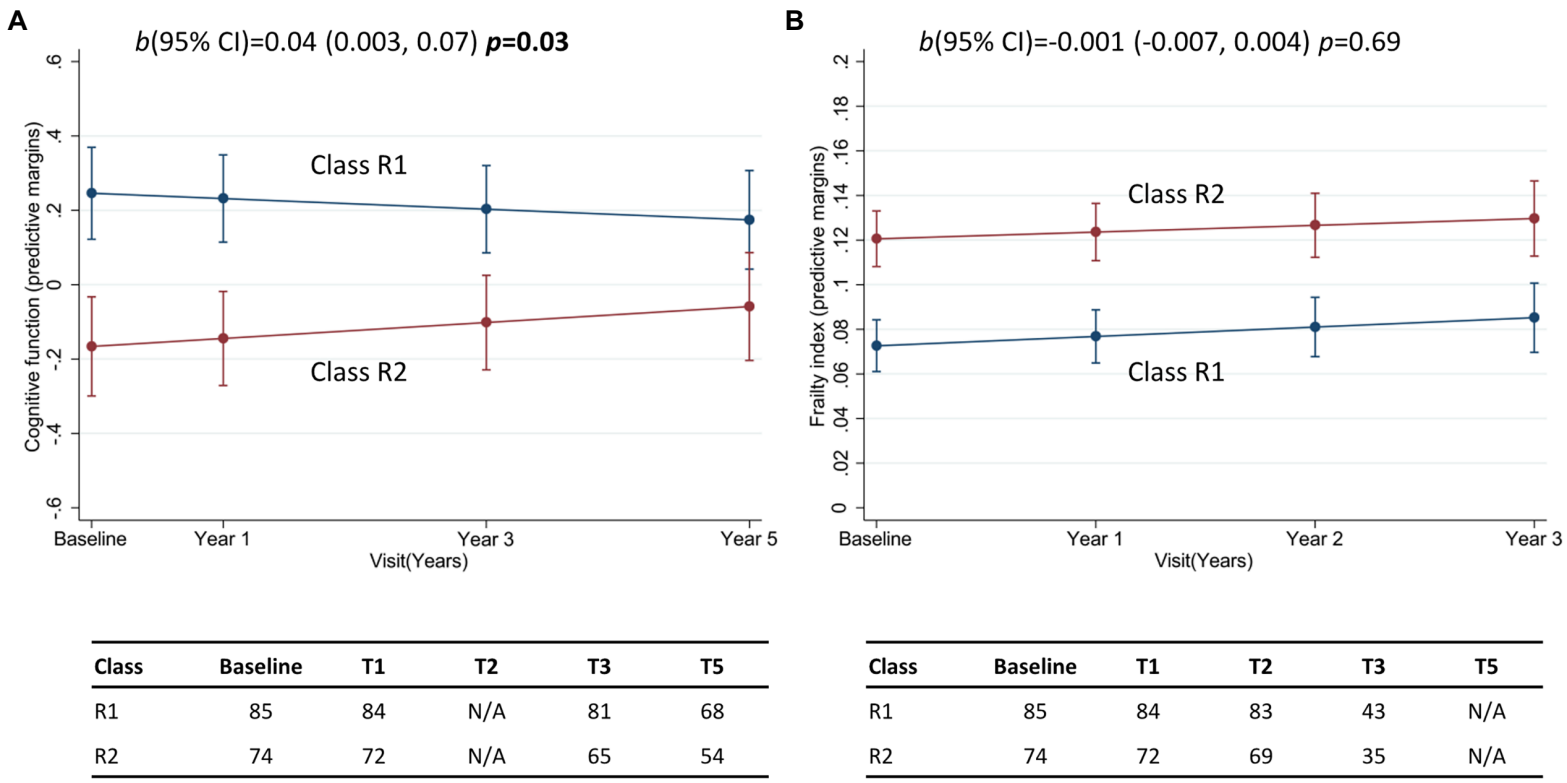
Margin plots presenting the two-way interaction between subclasses of the resilient brain age group and the longitudinal change in **(A)** composite cognitive function, and **(B)** frailty index. Tables report the frequency of participants who provide data at baseline, and at follow-up assessments. b(95% CI) = unstandardized beta-coefficient of the two-way interaction between class membership and time, and the corresponding 95% confidence interval.

**Figure 5:**
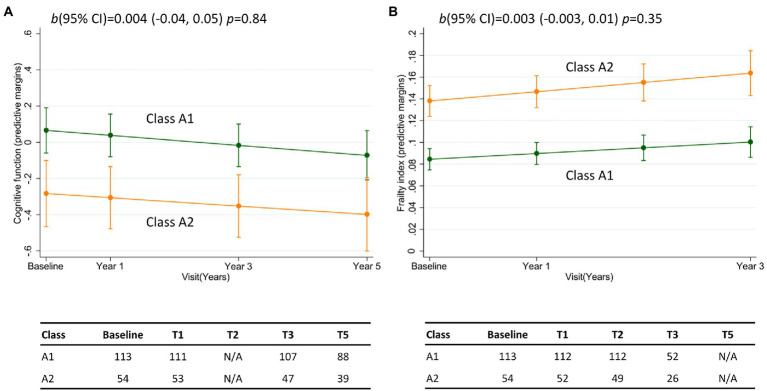
Margin plots presenting the two-way interaction between subclasses of the advanced brain age group and the longitudinal change in **(A)** composite cognitive function, and **(B)** frailty index. Tables report the frequency of participants who provide data at baseline, and at follow-up assessments. b(95% CI) = unstandardized beta-coefficient of the two-way interaction between class membership and time, and the corresponding 95% confidence interval.

Class membership was associated with longitudinal change in composite cognitive function for resilient brain agers (Table 2). Here was a relatively weak, though statistically significant positive interaction that was equivalent to a 0.04 standard deviation difference per year. We present this finding in Figure 4B, which shows class R1 had a better cognitive performance at baseline that decreased marginally over consecutive visits, while class R2 had a lower cognitive score that increased over the 5-year follow-up. Class membership was not significantly associated with change in FI (Table 2; Figures 4B). For the advanced brain age group, class membership was not significantly predictive of longitudinal change in composite cognitive function or FI (Table 2; Figures 5A,B).

**Table 2:** Linear mixed models examining the association between health-related profiles in brain aging and change in composite cognitive function and frailty index (FI).

	Unadjusted		Adjusted	
	^*a*^*b* (95% CI)	*p*	^*a*^*b* (95% CI)	*p*
**Resilient (n=159):**				
**Composite cognitive function**				
Class R2 vs R1	0.03 (−0.002, 0.06)	0.07	0.04 (0.003, 0.07)	0.03*
**FI**				
Class R2 vs R1	−0.001 (−0.01, 0.01)	0.84	−0.001 (−0.01, 0.004)	0.69
**Advanced (n=167):**				
**Composite cognitive function**				
Class R2 vs R1	0.01 (−0.04, 0.05)	0.78	0.01 (−0.04, 0.05)	0.84
**FI**				
Class R2 vs R1	0.003 (−0.003, 0.01)	0.33	0.003 (−0.003, 0.01)	0.33

*p < 0.05. Models were adjusted for chronological age, sex and education. ^a^Unstandardized beta-coefficient of the two-way interaction between class membership and time, which represents the difference in the rate of change in cognition and frailty between class 2 relative to class 1.

To confirm our main findings, latent classes were also identified in the full sample of participants, without brain age stratification (Supplementary Table 7). Class membership was predictive of longitudinal change in cognitive function (Supplementary Figure 1), whereby participants in class T1 started with a high cognitive performance that decreased over time, while class T2 had a lower cognitive score that continued to decrease over the 5 years. Critically, while cognition was comparable between groups of advanced and resilient brain agers, there was also a significant interaction between class membership and brain-PAD, consistent with our main observations (Supplementary Figure 1). People with resilient brain aging in class 1 had high composite cognitive function scores at baseline that decreased over time, while class 2 had lower cognitive function at baseline, but show an increase over 5 years. Conversely, people in both class 1 and 2 with advanced brain aging show a gradual decrease in cognitive performance over follow-up. Class membership was neither directly associated with FI, nor was an interaction with brain-PAD observed (Supplementary Figure 1).

## Discussion

In a cohort of relatively healthy, cognitively unimpaired older adults, this study used a combination of brain age prediction, data-driven clustering and predictive modelling to determine whether there is heterogeneity within *‘resilient’* and *‘advanced’* brain age groups related to health, behavioral or cognitive measures, and how this heterogeneity relates to future longitudinal trajectories of cognition and frailty. We first demonstrate that there is health-related heterogeneity in the older population that is independent of brain aging. This was summarized by two qualitatively similar health-related clusters being identified in each brain age group, one largely characterized by physical qualities (i.e., low prevalence of obesity and better physical health; Class 1), and the other by poor cardiometabolic health and cognitive function (Class 2). Class membership was predictive of longitudinal change in cognitive function only for resilient brain agers.

To our knowledge, this is the first study to explore whether there are health-related profiles of brain aging that differentially contribute to changes in cognitive and functional performance. While brain age provides a simple and interpretable measure for understanding deviations from normative aging trajectories, prior evidence suggests there may be multiple imaging signatures that effectively characterize brain aging in older adults (Eavani et al., 2018). Data-driven approaches have also identified considerable diversity in neurological diseases, which holds great promise for precision medicine (Habes et al., 2020; Wen et al., 2022).

Our findings suggest that cluster formation based on cardiometabolic, physical and cognitive factors appear to be a stable and generalizable feature of the older population. This aligns with established literature (Oldridge et al., 2001; Waldstein and Katzel, 2004; Sullivan et al., 2007; Lai et al., 2020), and findings from the larger ASPREE study (19,114 participants from United States and Australia; Phyo et al., 2021a). In both brain age categories, “cluster 1” was characterized by lower prevalence of obesity and better perceived physical QoL (i.e., defined by physical functioning, role-physical, bodily pain and general health; Ware et al., 1996), while “cluster 2” had a higher prevalence of hypertension, poorer physical QoL, and a lower general cognitive status.

Our finding that living alone and higher cognitive performance are linked to low levels of obesity and better physical health was confined to people with advanced brain aging. This was despite the comparable proportion of these factors in the resilient and advanced brain age groups. Here, discrepancies may partly reflect a benefit of independent living on cognitive function through flexible neural processes, even with increasing brain atrophy (i.e., cognitive reserve; Evans et al., 2019; Stern et al., 2020; Villa et al., 2021). Living alone is a common occurrence for older adults due to the natural shift in social environments with aging (Evans et al., 2019; Bzdok and Dunbar, 2020; Villa et al., 2021). Although this imposes a greater risk for social isolation and loneliness (Finlay and Kobayashi, 2018), there is some evidence to suggest older people who live alone are more likely to engage in regular social activities (Evans et al., 2019). Social engagement requires complex cognitive processes that may help improve reserve (Stern et al., 2020). While this remains speculative, people with advanced brain aging may have maintained a higher cognitive performance through the influence of independent living on cognitive reserve, but this warrants further investigation (Evans et al., 2019; Stern et al., 2020; Villa et al., 2021).

Class membership was predictive of longitudinal change in cognitive function for resilient brain agers only and remained when assessed in the total cohort. In this subgroup of resilient brain agers, individuals with greater cognitive ability and physical functioning at baseline were more likely to show cognitive decline over 5 years. Conversely, poorer cognitive function and physical health were associated with improvement on the cognitive tests, on average, over the study period. This was a small effect that may reflect a ‘regression to the mean’ (i.e., resilient brain agers with a high or low cognitive score at baseline will present a score that is closer to the mean at follow-up; Barnett et al., 2005). However, the analogous subgroup of people with relatively poorer cognition and physical health in the *advanced* brain age group did not demonstrate a similar effect; these individuals declined in performance over time at the same rate as those in the high-cognition subgroup. Therefore, these findings could indicate a residual capacity, or “brain reserve” (Stern et al., 2020) in the resilient brain age group. For individuals already functioning at or near to ceiling for their age group, the most likely longitudinal trajectories are stability or decline, whereas for people operating below capacity at baseline, opportunities for performance improvement due to learning (e.g., repeated practice), motivation, or intervention are available. This same reserve is not available to people with an advanced brain age. Investigating the qualitative differences in longitudinal growth trajectories will overcome this study’s limitation of identifying latent classes cross-sectionally, and further our understanding of these findings. Interestingly, some epidemiological studies have also reported a decelerated cognitive decline, and a reduced risk of incident dementia, in older adults at increased risk of metabolic disease, including a high body fat mass and hypertension (Van den Berg et al., 2007; Forti et al., 2010; Watts et al., 2013). Although this finding may be specific to individuals with early neurodegenerative disease, as opposed to the healthy population (Watts et al., 2013), emerging evidence suggests a higher body fat mass may actually be beneficial for maintaining cognitive function in older adults, especially women (Luchsinger et al., 2013; Bohn et al., 2020). A meta-analysis of six prospective community-based studies also identified a reduced risk of dementia when hypertension is controlled through anti-hypertensive medications (Ding et al., 2020); more than half of the current NEURO cohort with hypertension were on treatment for high blood pressure. Further, individuals who have a stable cardiovascular risk profile over 5 years have a reduced likelihood of dementia compared with those with increasing risk over time (D’Agostino Sr et al., 2008; Farnsworth von Cederwald et al., 2022). While there remains insufficient evidence to provide a clear interpretation of how different lifestyle and health factors influence aging trajectories, future prospective analysis of these health clusters may improve our understanding of the interaction between brain aging and cognitive outcomes in older adults.

This study has a number of strengths. Firstly, we derived estimates of brain age from MRI following a rigorous quality assessment and using a publicly sourced model that has been previously validated using an independent test cohort (Cole et al., 2018), and which we have previously shown is not biased by chronological age in our population (Wrigglesworth et al., 2022a). We also identified subclasses of people within the resilient and advanced brain age groups using a person-centered approach that focuses on identifying homogenous groups of people rather than homogeneous groups of variables (Muthén and Muthén, 2012). Further, our application of LCA allowed for quantification of uncertainty of class membership through posterior probabilities, and permitted the usage of mixed data types (Andreopoulos et al., 2009; Sinha et al., 2021). A robust approach to assessing model fit and classification accuracy was adopted through the combined use of multiple inferential (i.e., LR chi-square goodness of fit) and descriptive tools (i.e., AIC/BIC information criterion). Finally, phenotypic changes in frailty and cognitive function were investigated prospectively using data from a large community-based older cohort, focusing on a stage of life characterized by great heterogeneity of aging trajectories.

There are several limitations that must also be considered. First, the moderate correlation between brain age and chronological age for our study cohort was weaker than achieved for the held-out test (*n* = 857, *r* = 0.973) and validation cohorts (*n* = 611, *r* = 0.947; Cole et al., 2018). Here, a less than accurate fit of the model may relate to the narrow age-range of our study sample, which contrasts the broad age-range of the cohorts used to train and validate the model (18 to 92 and 90 years respectively). This is further supported by the larger mean error of prediction when adjusted for age-range (0.28 vs. 0.05 and 0.07 years for held-out and test datasets, respectively).

We identified latent classes cross-sectionally, rather than exploring the qualitative differences in longitudinal growth trajectories (Nylund et al., 2007; Eavani et al., 2018). General limitations of LCA include handling missing data, sensitivity to extreme outliers, and reliance on relatively large sample sizes for reliability (Mccutcheon, 1987; Nylund et al., 2007; Collins and Lanza, 2009; Berlin et al., 2014). We mitigated the influence of these limitations by evaluating multiple fit criteria and selecting variables with a relative frequency greater than 10% of the sample (Sinha et al., 2021). Our study sample included primarily white people who were generally healthy, clinically cognitively unimpaired [i.e., no dementia diagnosis or 3MS (Teng and Chui, 1987) score ≥ 78], and had completed 12 or more years of formal education, potentially limiting generalizability to the wider population (Masyn, 2013). Longitudinal study attrition may have included individuals with greater health deficits and poorer cognitive function. Lastly, given the modest number of participants within each class, we may not have been sufficiently powered to detect subtle interindividual differences in the longitudinal change in frailty index and cognitive function over the three and 5 year follow-up period, respectively (Sinha et al., 2021).

In conclusion, this study is the first to demonstrate heterogeneity related to health, behavior, cognitive function, quality of life within the construct of brain age prediction in an older community-dwelling cohort. Given the large spectrum of interacting factors in a real-world setting, it is critical to our understanding of brain age to consider the influence of biological and functional measures in aggregate. Identification of differences in health-related characteristics based on brain aging may help accurately predict long term outcomes, prevention and treatment strategies.

## Data availability statement

The original contributions presented in the study are included in the article/Supplementary material, further inquiries can be directed to the corresponding author.

## Ethics statement

The studies involving human participants were reviewed and approved by Monash University Human Research and Ethics Committee. The participants provided their written informed consent to participate in the ASPREE and NEURO studies, and study procedures were conducted in accordance with institutional guidelines.

## Author contributions

JW and IH contributed to the conception and design of the study. JW performed the statistical analysis and wrote the first draft of the manuscript. JR helped with the analysis and interpretation of the results. All authors contributed to the article and approved the submitted version.

## Funding

ASPREE received funding from the National Institute on Aging and the National Cancer Institute at the National Institutes of Health (U01AG029824), Monash University, the Victorian Cancer Agency, and the Australian National Health and Medical Research Council (NHMRC, grant numbers 334047 and 1127060). NEURO received funding from NHMRC (1086188) and support from Monash Biomedical Imaging. JW is the recipient of a Research Training Program stipend, awarded by Monash University and the Australian government. IH is supported by a NHMRC Fellowship (APP1106533). JR is funded by a NHMRC Dementia Research Leader Fellowship (APP1135727). Funders neither directed the conduction of this study, nor the decision to publish these findings.

## Conflict of interest

The authors declare that the research was conducted in the absence of any commercial or financial relationships that could be construed as a potential conflict of interest.

## Publisher’s note

All claims expressed in this article are solely those of the authors and do not necessarily represent those of their affiliated organizations, or those of the publisher, the editors and the reviewers. Any product that may be evaluated in this article, or claim that may be made by its manufacturer, is not guaranteed or endorsed by the publisher.

## Footnotes

^1^ https://github.com/james-cole/brainageR
