# Supplementary material for: Health-related heterogeneity in brain aging and associations with longitudinal change in cognitive function
Source: Front Aging Neurosci. 2023 Jan 4;14:1063721. doi: 10.3389/fnagi.2022.1063721 (PMC9846261; doi:10.3389/fnagi.2022.1063721)
Supplement: Supplementary file 1 [file Data_Sheet_1.docx]

Supplementary Material

# Supplementary Figures and Tables

**1.1. Supplementary tables**

**Supplementary Table 1.** Baseline participant characteristics comparing included and excluded study participants.

|  | **Included (n=326)** | | | **Excluded (n=205)** | |
| --- | --- | --- | --- | --- | --- |
| **Characteristics** | **Resilient (n=159)** | **Advanced (n=167)** | ***p*** |  | ***p*** |
| Age group |  |  | 0.33 |  | 0.84 |
| <75 | 120 (75.5) | 118 (70.7) |  | 148 (72.2) |  |
| 75+ | 39 (24.5) | 49 (23.3) |  | 57 (27.8) |  |
| Female gender, n (%) | 68 (42.8) | 70 (41.9) | 0.88 | 119 (58.1) | <0.0001* |
| <12 years education, n (%) | 103 (64.8) | 106 (63.5) | 0.81 | 87 (42.4) | 0.13 |
| ^b^Obese, n (%) | 50 (31.5) | 47 (28.3) | 0.54 | 48 (23.7) | 0.12 |
| Ever smoked, n (%) | 68 (42.8) | 74 (44.3) | 0.78 | 85 (41.5) | 0.64 |
| Lives alone, n (%) | 36 (22.6) | 47 (28.1) | 0.25 | 60 (29.3) | 0.34 |
| Hypertensive, n (%) | 119 (74.8) | 111 (66.5) | 0.1 | 141 (68.8) | 0.67 |
| 3MS overall score, n (%) |  |  |  |  |  |
| Tertile 1 (78-93) | 62 (39) | 60 (35.9) | 0.78 | 76 (37.1) | 0.03^*^ |
| Tertile 2 (94-96) | 58 (36.5) | 61 (36.5) |  | 56 (27.3) |  |
| Tertile 3 (97-100) | 39 (24.5) | 46 (27.5) |  | 73 (35.6) |  |
| Mental QoL, n (%) |  |  |  |  |  |
| Tertile 1 (28.5-54.7) | 50 (31.5) | 59 (35.3) | 0.72 | 68 (33.2) | 0.62 |
| Tertile 2 (54.7-59.3) | 56 (35.2) | 53 (31.7) |  | 64 (31.2) |  |
| Tertile 3 (59.4-78.8) | 53 (33.3) | 55 (32.9) |  | 73 (35.6) |  |
| Physical QoL, n (%) |  |  |  |  |  |
| Tertile 1 (16.2-46.6) | 53 (33.3) | 56 (33.5) | 0.98 | 61 (29.8) | 0.32 |
| Tertile 2 (46.8-54.0) | 55 (34.6) | 56 (33.5) |  | 70 (34.2) |  |
| Tertile 3 (54.0-63.7) | 51 (32.1) | 55 (32.9I) |  | 74 (36.1) |  |
| Frailty index, mean (SD) |  |  |  |  |  |
| Baseline | 0.10 (0.06) | 0.10 (0.06) | 0.26 | 0.10 (0.06) | 0.66^a^ |
| Year one | 0.10 (0.07) | 0.10 (0.07) | 0.58 | 0.10 (0.06) | 0.86^a^ |
| Year two | 0.10 (0.07) | 0.12 (0.08) | 0.11 | 0.11 (0.07) | 0.53^a^ |
| Year three | 0.10 (0.08) | 0.11 (0.07) | 0.08 | 0.10 (0.06) | 0.49^a^ |
| Composite cognitive function, mean (SD) |  |  |  |  |  |
| Baseline | 0.05 (0.68) | -0.05 (0.70) | 0.18 | 0.03 (0.68) | 0.38^a^ |
| Year one | 0.07 (0.62) | -0.09 (0.83) | 0.18 | 0.01 (0.73) | 0.62 |
| Year three | 0.06 (0.71) | -0.05 (0.71) | 0.20 | 0.08 (0.68) | 0.04* |
| Year five | 0.12 (0.67) | -0.12 (0.70) | 0.006^*^ | 0.09 (0.68) | 0.04* |

**p<0.05. ^a^Rank sum performed due to skewed distribution for either, or both resilient and advanced brain aging. ^b^Participants identified as ‘not obese’ include those with a body mass index defined according to the World Health Organization criteria as underweight(<20; resilient: n=0; advanced: n=3; excluded: n=2), normal (20-24.9; resilient: n=35; advanced: n=48; excluded: n=83) and overweight (25-29.9; resilient: n=74; advanced n=68; excluded: n=142) (Wolfe et al., 2018). Missing data for obesity (advanced: n=1; excluded: n=2), and anti-hypertensive medication data (resilient: n=6; advanced: n=10; excluded: n=5). Participants missing data for obesity at baseline (advanced: n=1; excluded: n=2); frailty at the one (resilient: n=3; advanced: n=3; excluded: n=4), two (resilient: n=7; advanced: n=6; excluded: n=5) and three year visit (resilient: n=81; advanced: n=89; excluded: n=99); composite cognitive function at the one (resilient: n=3; advanced: n=3; excluded: n=4), three (resilient: n=14; advanced: n=13; excluded: n=9) and five year visit (resilient: n=37; advanced: n=40; excluded: n=43). Abbreviations: QoL=quality of life.*

**Supplementary Table 2.** Unconditional latent class models for resilient and advanced brain aging.

|  | **Resilient (n=159)** | |
| --- | --- | --- |
| **Characteristics** | **Class 1 (54%)** | **Class 2 (47%)** |
| ^a^Obese | **0.09** | 0.56 |
| Ever smoked | 0.43 | 0.42 |
| ^a^Hypertensive | 0.60 | **0.91** |
| Lives alone | **0.21** | **0.25** |
| ^a^3MS overall score |  |  |
| Tertile 1 (78-93) | 0.33 | 0.45 |
| Tertile 2, (94-96) | 0.35 | 0.38 |
| Tertile 3, (97-100) | 0.32 | **0.17** |
| ^a^Mental QoL |  |  |
| Tertile 1 (28.5-54.7) | 0.35 | **0.28** |
| Tertile 2 (54.7-59.1) | 0.43 | **0.27** |
| Tertile 3 (59.1-78.8) | **0.22** | 0.45 |
| ^a^Physical QoL |  |  |
| Tertile 1 (16.2-46.6) | **0.15** | 0.53 |
| Tertile 2 (46.8-54.0) | 0.31 | 0.39 |
| Tertile 3 (54.0-63.7) | 0.54 | **0.08** |
| **AvePP** | 0.83 | 0.84 |
|  | **Advanced (n=167)** | |
|  | **Class 1 (68%)** | **Class 2 (32%)** |
| ^a^Obese | **0.14** | 0.62 |
| Ever smoked | 0.41 | 0.51 |
| ^a^Hypertensive | 0.57 | **0.89** |
| Lives alone | 0.39 | **0.01** |
| ^a^3MS overall score |  |  |
| Tertile 1 (78-93) | **0.27** | 0.58 |
| Tertile 2, (94-96) | 0.34 | 0.42 |
| Tertile 3, (97-100) | 0.39 | **3.52E-07** |
| ^a^Mental QoL |  |  |
| Tertile 1 (28.5-54.7) | **0.29** | 0.50 |
| Tertile 2 (54.7-59.1) | 0.39 | **0.14** |
| Tertile 3 (59.1-78.8) | 0.31 | 0.37 |
| ^a^Physical QoL |  |  |
| Tertile 1 (16.2-46.6) | **0.26** | 0.51 |
| Tertile 2 (46.8-54.0) | 0.35 | **0.30** |
| Tertile 3 (54.0-63.7) | 0.39 | **0.19** |
| **AvePP** | 0.93 | 0.78 |

*Bold:* *high class homogeneity, defined by a conditional probability* $\leq$*0.30 or* $\geq$*0.70 (Masyn, 2013). ^a^Indicates variables showing statistically significant association between exposure and class assignment, as per our results presented in Table 2. Abbreviations: AvePP=average class probability; QoL=quality of life.*

**Supplementary Table 3.** Measures of association conditional to the latent class identified for resilient brain aging, to examine assumption of local independence.

|  | **Class R1** | | | | | | |
| --- | --- | --- | --- | --- | --- | --- | --- |
|  | **Obese** | **Ever smoked** | **Hypertensive** | **Lives alone** | **3MS overall score** | **Mental QoL** | **Physical QoL** |
| **Obese** | 1.00 | -0.02 | 0.01 | 0.14 | 0.14 | 0.12 | 0.22 |
| **Ever smoked** | -0.02 | 1.00 | 0.03 | 0.002 | 0.18 | 0.08 | 0.04 |
| **Hypertensive** | 0.01 | 0.03 | 1.00 | -0.14 | 0.07 | 0.27 | 0.25 |
| **Lives alone** | 0.14 | 0.002 | -0.14 | 1.00 | 0.04 | 0.30 | 0.24 |
| **3MS overall score** | 0.14 | 0.18 | 0.07 | 0.04 | 1.00 | 0.09 | 0.16 |
| **Mental QoL** | 0.12 | 0.08 | 0.27 | 0.30 | 0.09 | 1.00 | 0.06 |
| **Physical QoL** | 0.22 | 0.04 | 0.25 | 0.24 | 0.16 | 0.06 | 1.00 |
|  | **Class R2** | | | | | | |
| **Obese** | 1.00 | 0.06 | -0.19 | -0.13 | 0.08 | 0.25 | 0.20 |
| **Ever smoked** | 0.06 | 1.00 | -0.04 | -0.09 | 0.18 | 0.32 | 0.20 |
| **Hypertensive** | -0.19 | -0.04 | 1.00 | 0.15 | 0.09 | 0.01 | 0.20 |
| **Lives alone** | -0.13 | -0.09 | 0.15 | 1.00 | 0.09 | 0.13 | 0.21 |
| **3MS overall score** | 0.08 | 0.18 | 0.09 | 0.09 | 1.00 | 0.12 | 0.10 |
| **Mental QoL** | 0.25 | 0.32 | 0.01 | 0.13 | 0.12 | 1.00 | 0.14 |
| **Physical QoL** | 0.20 | 0.20 | 0.20 | 0.21 | 0.10 | 0.14 | 1.00 |

*Abbreviations: QoL=quality of life.*

**Supplementary Table 4.** Measures of association conditional to the latent class identified for advanced brain aging, to examine assumption of local independence.

|  | **Class A1** | | | | | | |
| --- | --- | --- | --- | --- | --- | --- | --- |
|  | **Obese** | **Ever smoked** | **Hypertensive** | **Lives alone** | **3MS overall score** | **Mental QoL** | **Physical QoL** |
| **Obese** | 1.00 | 0.06 | -0.15 | 0.004 | 0.24 | 0.06 | 0.16 |
| **Ever smoked** | 0.06 | 1.00 | 0.15 | 0.13 | 0.17 | 0.17 | 0.06 |
| **Hypertensive** | -0.15 | 0.15 | 1.00 | 0.15 | 0.13 | 0.08 | 0.14 |
| **Lives alone** | 0.004 | 0.13 | 0.15 | 1.00 | 0.10 | 0.30 | 0.27 |
| **3MS overall score** | 0.24 | 0.17 | 0.13 | 0.10 | 1.00 | 0.11 | 0.14 |
| **Mental QoL** | 0.06 | 0.17 | 0.08 | 0.30 | 0.11 | 1.00 | 0.06 |
| **Physical QoL** | 0.16 | 0.06 | 0.14 | 0.27 | 0.14 | 0.06 | 1.00 |
|  | **Class A2** | | | | | | |
| **Obese** | 1.00 | 0.04 | -0.24 | N/A | 0.01 | 0.25 | 0.32 |
| **Ever smoked** | 0.04 | 1.00 | -0.19 | N/A | 0.04 | 0.12 | 0.12 |
| **Hypertensive** | 0.01 | 0.04 | 1.00 | N/A | -0.11 | 0.15 | 0.21 |
| **^a^Lives alone** | N/A | N/A | N/A | N/A | N/A | N/A | N/A |
| **3MS overall score** | 0.01 | 0.04 | -0.11 | N/A | 1.00 | 0.32 | 0.27 |
| **Mental QoL** | 0.25 | 0.12 | 0.15 | N/A | 0.32 | 1.00 | 0.21 |
| **Physical QoL** | 0.32 | 0.12 | 0.21 | N/A | 0.27 | 0.21 | 1.00 |

*^a^Results are N/A as there were zero participants answering ‘yes’ to living alone in Class A2. Abbreviations: QoL=quality of life.*

**Supplementary Table 5.** Demographic and lifestyle characteristics of the latent classes identified from the total cohort of resilient and advanced brain agers.

|  | **Total cohort (n=326)** | | |
| --- | --- | --- | --- |
| **Characteristics** | **Class T1 (57%)** | **Class T2 (43%)** | ***p*** |
| Age group |  |  | 0.19 |
| <74 | 141 (75.8) | 97 (69.3) |  |
| 75+ | 45 (24.2) | 43 (30.7) |  |
| Female gender, n (%) | 82 (44.1) | 56 (40.0) | 0.46 |
| <12 years education, n (%) | 62 (33.3) | 55 (39.3) | 0.27 |
| Obese, n (%) | 9 (4.9) | 88 (62.9) | <0.0001 |
| Ever smoked, n (%) | 76 (40.9) | 66 (47.1) | 0.26 |
| Hypertensive, n (%) | 101 (54.3) | 129 (92.1) | <0.0001 |
| Lives alone, n (%) | 61 (32.8) | 22 (15.7) | <0.0001 |
| 3MS overall score, n (%) |  |  | <0.0001 |
| Tertile 1 (78-93) | 51 (27.4) | 71 (50.7) |  |
| Tertile 2, (94-96) | 61 (32.8) | 58 (41.4) |  |
| Tertile 3, (97-100) | 74 (39.8) | 11 (7.9) |  |
| Mental QoL, n (%) |  |  | <0.0001 |
| Tertile 1 (28.5-54.7) | 54 (29.0) | 55 (39.3) |  |
| Tertile 2 (54.7-59.1) | 83 (44.6) | 26 (18.6) |  |
| Tertile 3 (59.1-78.8) | 49 (26.3) | 59 (42.1) |  |
| Physical QoL, n (%) |  |  | <0.0001 |
| Tertile 1 (16.2-46.6) | 34 (18.3) | 75 (53.6) |  |
| Tertile 2 (46.8-54.0) | 60 (32.3) | 51 (36.4) |  |
| Tertile 3 (54.0-63.7) | 92 (49.5) | 14 (10.0) |  |

*Bold=high class homogeneity, defined by a class-item probability* $\leq$*0.30 or* $\geq$*0.70 (Masyn, 2013). Hypertensive individuals reporting anti-hypertensive treatment (Class T1: n=56 [33%]; Class T2: n=96[69%]). Missing data for obesity (Class T1: n=1), and anti-hypertensive medication data (Class T1: n=15; Class T2: n=1) at baseline. Abbreviations: 3MS=global cognitive function measured using Modified-Mini-Mental State (3MS) examination score (Teng and Chui, 1987); QoL=quality of life.*

**Supplementary Table 6.** Unconditional latent class models for total cohort of resilient and advanced brain aging.

|  | **Total cohort (n=326)** | |
| --- | --- | --- |
| **Characteristics** | **Class T1 (57%)** | **Class T2 (43%)** |
| ^a^Obese | **0.10** | 0.55 |
| Ever smoked | 0.39 | 0.50 |
| ^a^Hypertensive | 0.57 | **0.88** |
| ^a^Lives alone | 0.31 | **0.18** |
| ^a^3MS overall score |  |  |
| Tertile 1 (78-93) | **0.28** | 0.49 |
| Tertile 2, (94-96) | 0.34 | 0.39 |
| Tertile 3, (97-100) | 0.37 | **0.12** |
| ^a^Mental QoL |  |  |
| Tertile 1 (28.5-54.7) | **0.29** | 0.39 |
| Tertile 2 (54.7-59.1) | 0.43 | **0.22** |
| Tertile 3 (59.1-78.8) | **0.28** | 0.40 |
| ^a^Physical QoL |  |  |
| Tertile 1 (16.2-46.6) | **0.21** | 0.49 |
| Tertile 2 (46.8-54.0) | 0.33 | 0.36 |
| Tertile 3 (54.0-63.7) | 0.46 | **0.15** |
| **AvePP** | 0.83 | 0.78 |

*Bold:* *high class homogeneity, defined by a conditional probability* $\leq$*0.30 or* $\geq$*0.70 (Masyn, 2013). ^a^Indicates variables showing statistically significant association between exposure and class assignment, as per our results presented in Supplementary Table 5. Abbreviations: AvePP=average class probability; QoL=quality of life.*

**Supplementary Table 7.** Linear mixed models examining the association between health-related profiles in brain aging and change in composite cognitive function and frailty index (FI).

|  | **Unadjusted** | | **Adjusted** | |
| --- | --- | --- | --- | --- |
| **Total cohort (n=326)** | ***^a^b* (95% CI)** | ***p*** | ***^a^b* (95% CI)** | ***p*** |
| **Composite cognitive function** |  |  |  |  |
| Class T2 vs T1 | 0.04 (0.001, 0.08) | 0.05* | 0.04 (0.003, 0.08) | 0.04* |
| Advanced vs Resilient | -0.01 (-0.04, 0.03) | 0.72 | -0.01 (-0.04, 0.03) | 0.74 |
| Interaction: Class T2 & Advanced | -0.05 (-0.10, 0.002) | 0.06 | -0.06 (-0.11, -0.002) | 0.04* |
| **FI** |  |  |  |  |
| Class T2 vs T1 | 0.003 (-0.003, 0.01) | 0.28 | 0.003 (-0.003, 0.01) | 0.35 |
| Advanced vs Resilient | 0.004 (-0.001, 0.01) | 0.11 | 0.004 (-0.001, 0.01) | 0.13 |
| Interaction: Class T2 & Advanced | -0.003 (-0.01, 0.01) | 0.49 | -0.002 (-0.01, 0.01) | 0.57 |

**p<0.05. Models were adjusted for chronological age, sex and education. ^a^Unstandardized beta-coefficient of a two-way interaction between class membership (‘Class T2 vs T1’) and time, or brain-PAD (‘Advanced vs Resilient’) and time, and is interpreted as the group difference in the rate of change in cognition and frailty. ‘Interaction: Class T2 & Advanced’ reports a three-way interaction between class membership, brain-PAD and time, and represents the difference in the rate of change in cognition and frailty between subclasses of brain agers with resilient and advanced brain aging.*

**1.1. Supplementary figures**

**Supplementary Figure 1.** Margin plots presenting the interaction between subclasses of health (A, D), or brain-predicted age difference (brain-PAD; B, E), and their interaction (C, F) with the longitudinal change in composite cognitive function and frailty index in the total cohort.*Footnote: T1 & T2 define health-related subclasses identified through a latent class analysis of the total sample; R1 & R2/A1 & A2 define subgroups of resilient or advanced brain agers in class 1 or 2, respectively.*


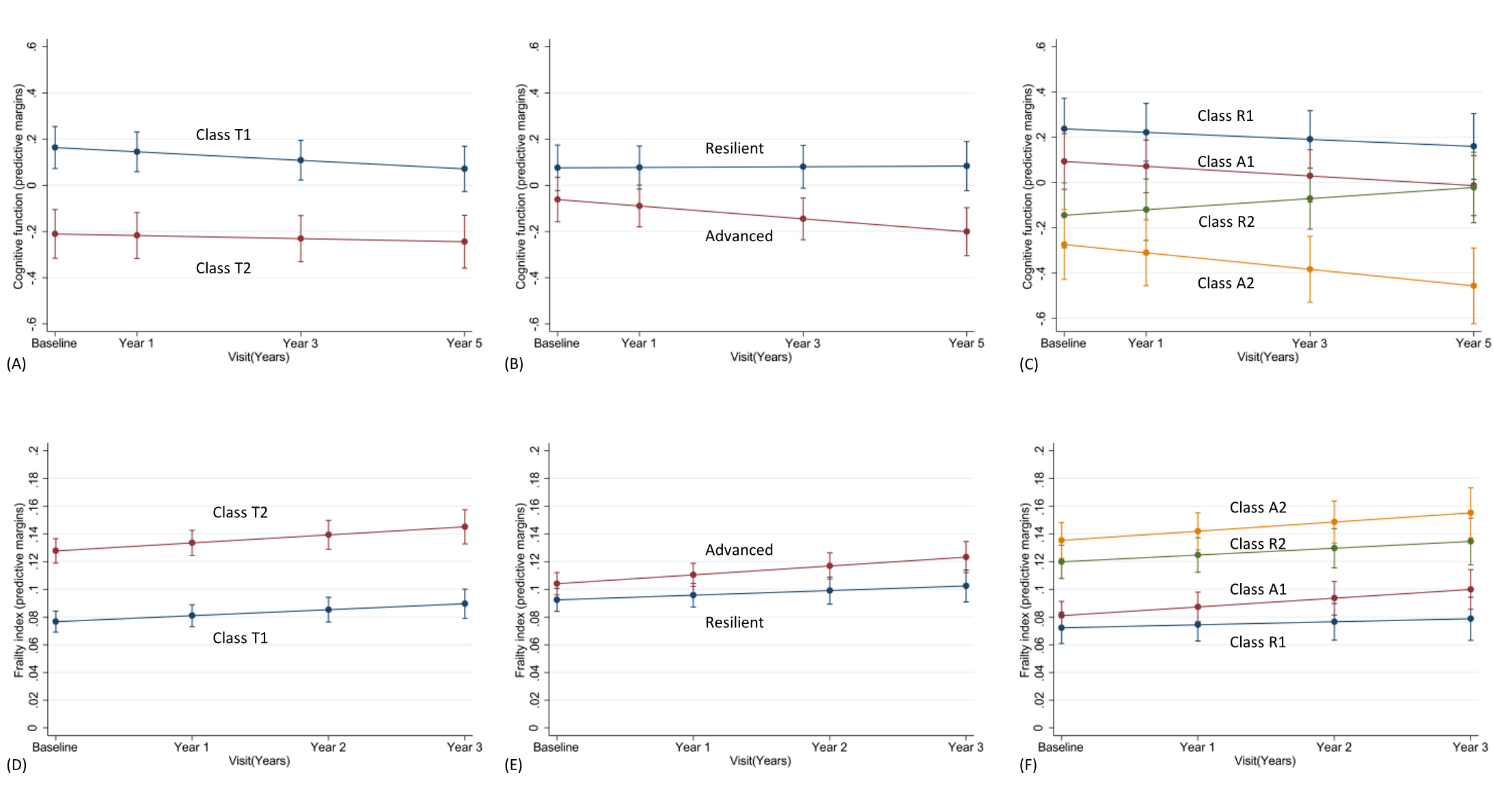


# References

MASYN, K. E. 2013. Latent Class Analysis and Finite Mixture Modeling. *In:* LITTLE, T. D. (ed.) *The Oxford Handbook of Quantitative Methods in Psychology: Statistical Analysis.* Oxford: Oxford University Press.

TENG, E. L. & CHUI, H. C. 1987. The Modified Mini-Mental State (3MS) examination. *J Clin Psychiatry,* 48**,** 314-8.

WOLFE, R., MURRAY, A. M., WOODS, R. L., KIRPACH, B., GILBERTSON, D., SHAH, R. C., NELSON, M. R., REID, C. M., ERNST, M. E., LOCKERY, J., DONNAN, G. A., WILLIAMSON, J. & MCNEIL, J. J. 2018. The aspirin in reducing events in the elderly trial: Statistical analysis plan. *Int J Stroke,* 13**,** 335-338.
